# Authenticity as Best-Self: The Experiences of Women in Law Enforcement

**DOI:** 10.3389/fpsyg.2022.861942

**Published:** 2022-05-06

**Authors:** Rochelle Jacobs, Antoni Barnard

**Affiliations:** Department of Industrial and Organisational Psychology, University of South Africa, Pretoria, South Africa

**Keywords:** authenticity, best-self, women, law enforcement, eudaimonic wellbeing, subjective wellbeing, hermeneutic phenomenology, identity work

## Abstract

Law enforcement poses a difficult work environment. Employees’ wellbeing is uniquely taxed in coping with daily violent, aggressive and hostile encounters. These challenges are compounded for women, because law enforcement remains to be a male-dominated occupational context. Yet, many women in law enforcement display resilience and succeed in maintaining a satisfying career. This study explores the experience of being authentic from a best-self perspective, for women with successful careers in the South African police and traffic law enforcement services. Authenticity research substantiates a clear link between feeling authentic and experiencing psychological wellbeing. The theoretical assumption on which the study is based holds that being authentic relates to a sense of best-self and enables constructive coping and adjustment in a challenging work environment. A qualitative study was conducted on a purposive sample of 12 women, comprising 6 police officers and 6 traffic officers from the Western Cape province in South Africa. Data were gathered through narrative interviews focussing on experiences of best-self and were analysed using interpretive phenomenological analysis. During the interviews, participants predominantly described feeling authentic in response to work-related events of a conflictual and challenging nature. Four themes were constructed from the data to describe authenticity from a best-self perspective for women in the study. These themes denote that the participating women in law enforcement, express feeling authentic when they present with a mature sense of self, feel spiritually congruent and grounded, experience self-actualisation in the work–role and realign to a positive way of being. Women should be empowered towards authenticity in their world of work, by helping them to acquire the best-self characteristics needed for developing authenticity.

## Introduction

To work in law enforcement is inherently stressful, rendering law enforcement officers particularly vulnerable when it comes to their psychological wellbeing (Lees et al., 2019; Bowen and Witkiewitz, 2020). Law enforcers suffer a plethora of physical and psycho-social stressors because, in the context of their work, they are frequently exposed to danger, violence, suffering, aggression, conflict and various physical and interpersonal threats (Anderson et al., 2002). The coping capacity and resilience of law enforcement employees are therefore consistently taxed, with potential negative consequences to service delivery and organisational effectiveness (Purba and Demou, 2019). Coping with such stress is made possible by manifesting one’s best-self, as characterised in being authentic (Roberts et al., 2005; Roche, 2010; Spreitzer et al., 2021). As such, this article focusses on authenticity from a best-self perspective in the context of women working in law enforcement. Activating best-self helps employees to feel authentic and flourish in the work place (Cable et al., 2015; Bakker and van Woerkom, 2018). In this study, authenticity is approached from a best-self perspective since it is rooted in the strength-based assumption underlying positive psychology, regarding best-self as a significant intrapsychic strength resource for resilience and flourishing in challenging and stressful work settings (Van Woerkom et al., 2020; Spreitzer et al., 2021), such as the context of this study.

While all who work in law enforcement face challenges to their wellbeing, these challenges are compounded for women, because law enforcement remains to be a male-dominated occupational context (Kurtz, 2012; Chen, 2015; McGinnis, 2019; Neely, 2019; Froehlich et al., 2020). Women encounter unique psychological stressors in male-dominated professions (Du Plessis and Barkhuizen, 2015; Indiana University, 2015; Gaines, 2017; Mayer et al., 2018). These stressors may lead to negative health outcomes for women (Indiana University, 2015) and make it more difficult for them to achieve the same success as men (Blackmore, 2017). Studies specific to law enforcement, have shown women to experience higher levels of stress than men because they encounter occupational barriers related to gender role stereotypes and biased social constructions of femininity (Kurtz, 2008, 2012; Chen, 2015). To adjust to gender-biased work cultures typical to male dominated work contexts, the coping behaviour of women can be related to the dynamics of identity work. Working women engage in identity work in order to remain authentic or true to themselves (Haeruddin, 2016). Women constantly negotiate the identity tensions that result from the inconsistencies they experience between the expectations of their stereotypical social role identity and their work role identity (Davey, 2008). Löve et al. (2011) similarly highlight the existential ambiguity that women experience because to cope in male-dominated occupations, they constantly compare the self against ambiguous and overwhelming life-role opportunities and expectations. It is not surprising that women have been reported to adopt characteristics and strategies not particular to who they are, to cope in such work environments (Du Plessis and Barkhuizen, 2015; Gaines, 2017). The demands of working in male-dominated occupations render the ability for women to be authentic difficult (Haeruddin, 2016; Jackson, 2019) and some women experience a lack of voice in such work places (Gaines, 2017). Still, many women in male-dominated occupations are resilient and persevere despite socio-psychological barriers and present with confidence and self-efficacy (Gaines, 2017). This is also true of many women in law enforcement who succeed in maintaining a satisfying career and seem to do and cope well in this environment.

Seminal work by Ménard and Brunet (2011) highlights the value of authenticity at work as a way of advancing employee wellbeing. The body of research linking authenticity to various types of wellbeing in the workplace is growing and signifies the essential role thereof in coping with challenging or difficult work circumstances (Ariza-Montes et al., 2019; Sutton, 2020). Authenticity is directly and indirectly linked to subjective wellbeing constructs such as life satisfaction (Vainio and Daukantaitė, 2016; Hwang and Kim, 2019) and engagement (Van den Bosch et al., 2019; Ortiz-Gómez et al., 2020; Sutton, 2020). Authenticity is essential to understanding adaptive characteristics pertaining to optimal self-esteem (Kernis, 2003; Kernis and Goldman, 2006) and employees who experience high levels of authenticity are strongly intrinsically motivated (Van den Bosch and Taris, 2018a,b) and exhibit self-regulatory and goal-directed behaviour (Chen and Murphy, 2019). Authenticity has also been shown to negatively relate to turnover intention (Ogruk and Anderson, 2018) and positively relate to work engagement, job satisfaction and performance (Metin et al., 2016). As such, authenticity has increasingly been regarded as an indicator of functioning well, coping constructively and adjusting optimally in the workplace.

Career development theories pertinent to women, isolate authenticity as a unique need in the career trajectories of women and fundamental to their work wellbeing (Barnard, 2018; Mainiero and Gibson, 2018; O’Neill and Jepsen, 2019). For women, feeling authentic is important to life satisfaction and coping with emotional burdens (Hwang and Kim, 2019). Using authenticity as a checkpoint for wellbeing, and because women’s authenticity experience is fundamental to their coping and flourishing in male-dominated work settings (Jackson, 2019), it is important to understand the authenticity experience of women. Flowing from a broader doctoral study on women developing authenticity (Jacobs, 2018), the objective of this study is to describe authenticity from a best-self perspective as experienced by women in law enforcement. To do so, the researchers explored narratives of best-self^1^ experiences to construct an understanding of what it means for them to feel authentic. This article contributes to understanding women’s ability to cope and flourish in the demanding law enforcement work context and inform developmental interventions to enhance their sense of authenticity and general wellbeing in a typical male-dominated occupation.

### Authenticity From a Best-Self Perspective

A clear and consistent empirical conceptualisation of the construct authenticity seems to remain elusive (Kreuzbauer and Keller, 2017; Baumeister, 2019; Lehman et al., 2019). Most definitions of authenticity follow the original idea of being that self that one truly is (Rogers, 1961) and incorporate the balancing of two ideals namely *feeling* and *acting* in congruence with one’s internal sense of self (Hewlin et al., 2020). Authenticity therefore denotes an *internal sense of self*. Ideas about what that means, are however multiple. Most commonly authenticity has been related to being true to the *core* self (Kernis, 2003), the *true* self (Kernis and Goldman, 2006; Schmader and Sedikides, 2018) or the *real* self (Hewlin et al., 2020; Sutton, 2020). Perspectives of being true to a *whole* self (Glavas, 2016), the *spiritual* self (Kiesling et al., 2006), *ideal* self (Vainio and Daukantaitė, 2016) or being true to one’s *best-self* (Roberts et al., 2005; Cable et al., 2013; Goodwin, 2019) have also been used to define authenticity.

From a paradigmatic point of view, two orientations frame the understanding of authenticity. The essentialist orientation views it as a process of self-discovery, while the existentialist view emphasises authenticity as a self-creation process (Sutton, 2020). Essentialists have promoted the idea of trait authenticity or personality trait consistency, seeking traits that align to a core or true self and seeking consistency in the expression and demonstration of one’s personality traits. Existentialists support the notion of state authenticity and view authenticity as a process of coherence or congruence, emphasising the extent to which one’s behaviour is self-determined and expressive of an evolving and integrated self (Sutton, 2020). In this view, being authentic allows for self-adjustment and change in a bigger context of integrating seemingly contradictory behaviours into a coherent self-concept (Harter, 2002). It also emphasises the personal agency of the individual in creating the self (Roberts et al., 2005). Behaviour is recognised as malleable and self-determined, and the self-integration process serves the pursuit of satisfying higher intrinsic goals or standards of being (Sheldon and Kasser, 1995) so that feeling authentic is expressive of the extent to which one feels aligned to one’s ideal self (Lenton et al., 2013). The extent of alignment between the ideal self and the current self has been referred to as the space of the best-self (Boyatzis and Dhar, 2021) acknowledging that expressing one’s best-self allows others to sense your true self more accurately (Human et al., 2012). State authenticity entails a self-verification process and implies an internal referent or benchmark against which one evaluates the extent of being true or congruent and results in a concomitant emotion (Lehman et al., 2019). Verification either results in positive emotions (high perceived congruence) or negative emotions (low perceived congruence), leading researchers to link authenticity with hedonic notions of subjective wellbeing (Ariza-Montes et al., 2017). Whereas research reporting the relationship between authenticity and subjective wellbeing abound, there is a lack of research exploring authenticity in the context of eudaimonic wellbeing (Sheldon, 2013). Hedonic wellbeing focusses on positive affect and life satisfaction, whereas eudaimonic wellbeing emphasise a fulfilling life based on the development of one’s best potentials. Authenticity in an eudaimonic sense implies the realisation of one’s best-self and the pursuit of actualising one’s full potential rather than pursuing feelings of pleasure (Pancheva et al., 2021). Authenticity as an eudaimonic concept has been said to integrate trait and state perspectives emphasising the reciprocity between feeling self-congruent and behaving consistently (Smallenbroek et al., 2017).

Earlier conceptual frameworks of authenticity emphasise the complexity of the concept and explain that being authentic involves self-awareness and understanding, processing of self-relevant evaluative information, behavioural consistency with one’s values and norms and open relational functioning (Kernis and Goldman, 2006). The tri-dimensional framework of Wood et al. (2008) have become popular and conceptualise authenticity as a disposition defined by one’s ability to follow and live according to one’s true emotions and values (authentic living); perceived congruence between your conscience and actual experience (low self-alienation); and one’s ability to resist external influences and expectations. In terms of the latter and third construct in the Wood et al. (2008) framework, research advancing the state authenticity concept has emphasised that authenticity is related to being open to external influence, rather than rejecting or resisting it (Sedikides et al., 2019). In this way, the state approach again emphasises personal agency in deciding who and how one is and wants to be (Roberts et al., 2005). Gecas (2000, 2003) similarly describes authenticity in agentic terms as a self-motive but emphasises that construction of the self is linked to social and cultural norms and ideologies of how one ought to be. The self-verification process is applied as an enactment of this self-motive and is based on the need to affirm or verify valued aspects of the self, which derives from value-driven social systems of meaning (Gecas, 2000, 2003; Roche, 2010).

The self-verification process that is integral to feeling authentic is thus implicitly influenced by a person’s engrained cultural norms and values of being good or living a good life (Rivera et al., 2019). This view acknowledges the idea of a socially constructed self and emphasises that authenticity is an individual experience with strong socially constructed ideals contributing to how a person judges the self. Barnard and Simbhoo (2014) define authenticity as an affective state, stemming from an ongoing self-appraisal of the degree to which self-expression corresponds with one’s subjective, socially formed expectation of the self, relative to others. This self-appraisal or self-verification is of an ongoing nature as authenticity is never an ultimately reachable end-state, it is relative to context, non-dualistic (not either-or) and dialectic (integrating contradictory aspects in the self; Roche, 2010). In the self-verification process, Baumeister (2019) regards authenticity as reflective of the extent to which individuals’ self-appraisal aligns with their *desired reputation*. In this vein, authenticity is a desirable state (Lehman et al., 2019) and usually reflect people’s idealistic view of self (Vainio and Daukantaitė, 2016) in that people seem to feel most authentic when they judge their actions and expressions as close to their ideal self (Lenton et al., 2013). This also reminds of Kernis and Goldman’s (2006) view of authenticity as reflecting optimal self-esteem (feeling of high self-worth and acceptance) and relates to Goodwin’s (2019, p. 33) use of best-self as the ‘self-checking’ point for women’s sense of authentic leadership. As such, in this study authenticity is defined from an eudaimonic, existential, state perspective and define it as an individual difference construct (Kernis, 2003; Wood et al., 2008) formed by an ongoing process (Barnard and Simbhoo, 2014) of the realignment of thoughts, emotions and behaviour (Roberts et al., 2005; Kernis and Goldman, 2006) with notions of best-self (Roberts et al., 2005; Human et al., 2012). Best-self denotes people’s cognitive construction of the qualities and characteristics they display when they deem themselves at their best (Roberts et al., 2005; Cable et al., 2013). Best-self is further regarded as an intrapsychic strength resource, a potential of being that already exists in the individual and to which one must realign to Padgett (2007) and Heidegger (2010), activate (Van Woerkom et al., 2020) and purposefully develop (Roberts et al., 2005). Goodwin (2019) draws a useful distinction between doing your best and being your best-self. Doing your best may be determined by others’ expectations and standards, while being your best-self is related to how you measure yourself in what you do (Goodwin, 2019).

## Methods

### Research Approach

This study followed a hermeneutic phenomenological approach, located in Heidegger’s hermeneutic ontology and interpretivist epistemology (Suddick et al., 2020). Heidegger’s ontology highlights the complex nature of reality that results from the entanglement between subjective being and a pre-existing world of meaning (Davidsen, 2013; Suddick et al., 2020). The intention of hermeneutic phenomenological research is to interpret lived experience and explain the meaning thereof (Horrigan-Kelly et al., 2016) in relation to a research phenomenon (Churchill, 2018). Interpretation involves uncovering meaning through intentional and reflective research acts (Suddick et al., 2020) integrated with preconceived frameworks of knowing (Davidsen, 2013; Horrigan-Kelly et al., 2016; Gyollai, 2020). Findings reflect a co-construction of what it means to be and feel authentic for the participating women, as framed in the preconceived eudaimonic notion of authenticity as best-self.

### Participants and Sampling

Purposeful sampling was employed because it suits the study’s exploratory aim (Saumure and Given, 2008) and selects participants who are able and willing to provide rich experiential information needed to best achieve the research aim (Durrheim and Painter, 2006; McMillan and Schumacher, 2010). In purposeful sampling criteria for inclusion are predetermined to ensure information-rich data sources with personal experience that is significant and relevant to the research objective (Leedy and Ormrod, 2010). The sampling method and inclusion criteria thus guided the identification of women in law enforcement, who could share rich information about their experiences of best-self. Tenure was deemed an important criterion to ensure data adequacy in the context of having had best-self experiences in the stressful work setting. Women with a sustained career of minimum of five years in law enforcement were therefore targeted. Based on the primary researcher’s previous employment in law enforcement, participants were accessed through mutual friends, ex-colleagues and approached *via* telephone or e-mail. The study ultimately included a purposive sample of 12 women working in law enforcement (6 police officers and 6 traffic officers) constituting a sample size well within the expected boundaries of a hermeneutic phenomenological study (Pietkiewicz and Smith, 2014; Alase, 2017). Such a sample size is appropriate in exploratory studies seeking to unearth rich concepts (McMillan and Schumacher, 2010), and in which multiple interviews are carried out with a participant (Riessman, 2008), to substantiate ideas of both the researcher and the participants (McMillan and Schumacher, 2010). In line with Malterud et al. (2016), the sampling strategy benefited this study by having information power, changing the focus to new knowledge produced by the analysis as opposed to participant numbers. Accordingly, the more information held by the sample that are of relevance to the research aim, the less participants are required. Tenure, position and demographic characteristics of the participants are summarised in Table 1.

**Table 1:** Descriptive profile of the research participants.

Participant	Age category (years)	Race	Level of work	Tenure (years)	Marital status	Children
PO1	46–55	Mixed race	Commissioned officer	25–30	Married	Yes
PO2	46–55	Mixed race	Commissioned officer	25–30	Single (D)	Yes
PO3	46–55	Black	Commissioned officer	11–15	Single	Yes
PO4	46–55	Black	Commissioned officer	25–30	Married	No
PO5	46–55	White	Commissioned officer	31–35	Single	Yes
PO6	36–45	Mixed race	Non-commissioned officer	5–10	Single	No
TO1	26–35	Black	Functional	5–10	Single (D)	Yes
TO2	26–35	Mixed race	Functional	5–10	Married	Yes
TO3	36–45	Mixed race	Functional	11–15	Married	Yes
TO4	36–45	Mixed race	Supervisory	11–15	Married	Yes
TO5	36–45	White	Functional	11–15	Single (D)	No
TO6	26–35	Mixed race	Functional	5–10	Single	No

PO, police officer; TO, traffic officer; D, divorced.

### Data Collection

During the course of 2017, narrative, face-to-face interviews were conducted because personal narratives are constitutive of identity and elicit notions of the construction and development of the self (Adler et al., 2017). Narrative interviews are unstructured, in-depth interviews that focus on eliciting lived stories about the research phenomenon in the context of past and present events or across time (Muylaert et al., 2014). Literature informed how the questions were structured (McMillan and Schumacher, 2010). Directed by the focus on authenticity as best-self, a core question was asked namely, ‘Share with me times you found it easier to be your best-self at work and times you found it more difficult.’ Elements in the integrative definition of authenticity from a best-self perspective were further considered, such as how aspects of emotions, thoughts, behaviour and strength(s) were experienced. Possible probing questions therefore included ‘How do thoughts and emotions influence your dealing with these challenges?’ and ‘So where do/did you find the strength to handle that?’ These, together with active listening ensured a natural conversation flow and an engaged narration to evolve during the interviews (Slembrouck, 2015). Follow-up interviews were conducted with all participants during which each of the women viewed the verbatim transcription of her initial interview. The purpose was to confirm and clarify where required, their initial contributions (McMillan and Schumacher, 2010), while the verbatim text was used as a prompt for them to elaborate or add experiences of best-self.

### Data Analyses

Congruent to hermeneutic phenomenology, data were analysed using interpretative phenomenological analysis (IPA; Smith, 2004; Horrigan-Kelly et al., 2016; Gyollai, 2020). Transcribed interviews constituted the primary data, which were deconstructed after repeated reading and familiarisation, by making descriptive notes relating the meaning of noteworthy pieces of data (Alase, 2017; Gyollai, 2020). Then, still attending closely to the participants’ verbatim narrative, descriptive notes that are conceptually related were categorised into clusters of meaning forming preliminary themes to describe authenticity. Refining and revising the themes ultimately lead to forming a framework of related superordinate themes and subthemes giving meaning to the research phenomenon (Pietkiewicz and Smith, 2014). The analytic process underlying IPA reflects a double hermeneutic process of interpretation, integrating participants’ making meaning of their experience and the researchers’ understanding of that meaning (Pietkiewicz and Smith, 2014; Alase, 2017). Credibility of the data analysis was enhanced by following a structured and rigorous data analysis method (Smith, 2004). The quality criteria proposed by Patton (1999) were applied by (i) using a method that is congruent to the methodological orientation of the study; (ii) applying analyst triangulation by subjecting the data to analysis from both authors in a consecutive and iterative manner; and (iii) using theory triangulation by critically interpreting data against existing literature conceptualising authenticity from an eudaimonic, state and best-self perspective.

Researcher reflexivity consistently guided the whole research process, specifically the data analysis stage. During the study and especially during data analysis, the researchers regularly cross-questioned their own experiences, specifically by frequently considering how their predispositions as women who previously worked in male-dominated settings, influenced their interpretations and meaning-making. With the primary researcher having worked in law enforcement herself, the secondary researcher assumed the role of seeking out data that challenged potential preconceived assumptions and values about how it is possible to be authentic in this context.

### Ethics Statement

Ethical clearance was obtained from the University of South Africa (Reference: 2016CEMS/IOP085). Participants gave informed consent for participation and recording of interviews, after being duly notified of the purpose and nature of the study and strategies to ensure protection of their rights to privacy, confidentiality and no harm. Pseudonyms as depicted in Table 1, are used to report on verbatim data in the findings.

## Findings

**Figure 1:**
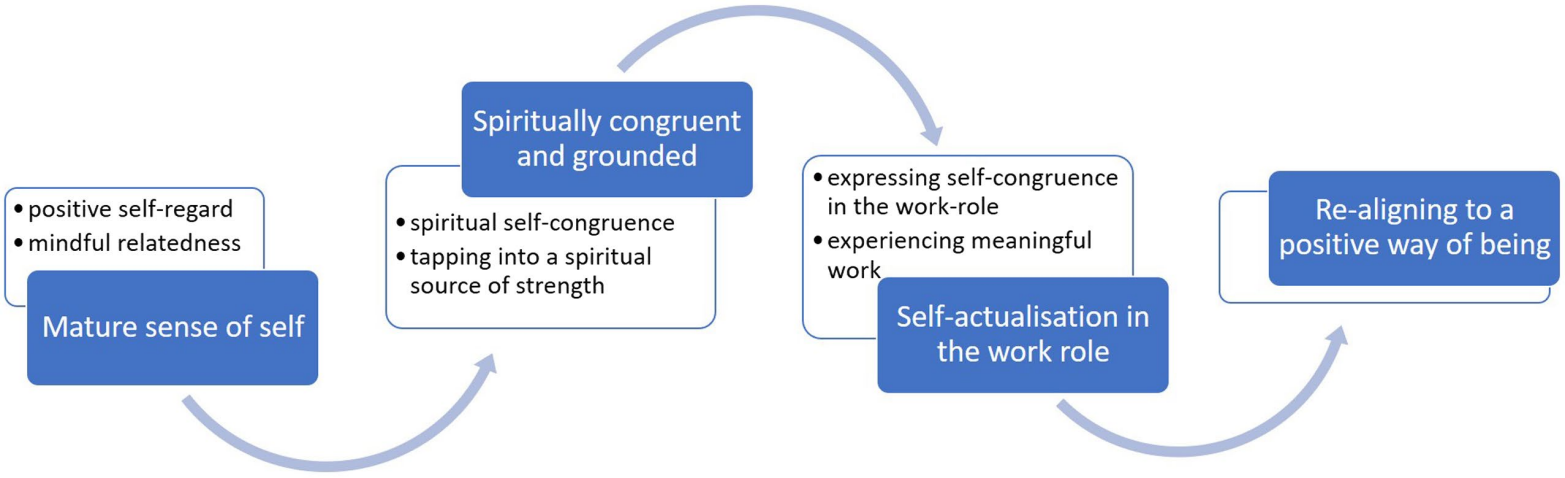
Themes describing the experience of authenticity as best-self.

Four superordinate themes were constructed to describe participants’ best-self experiences namely (i) a mature sense of self; (ii) feeling spiritually congruent and grounded; (iii) self-actualisation in the work role and (iv) realigning to a positive way of being. The four themes and their related subthemes are depicted in Figure 1 and described below, grounded in verbatim text from the data.

### A Mature Sense of Self

Participants narrate best-self experiences in relation to the extent to which they experience positive self-regard and express a mindful relatedness to others. These subthemes were clustered to describe a mature sense of self, which fundamentally describes balancing self-interest with the needs and interests of others. A mature sense of self is conceptualised as valuing the self in the awareness of limitations and strengths and mindfully relating to others through compassion and understanding of differences. A mature sense of self helps these women to cope with conflictual situations or people and experience a sense of best-self.

#### Positive Self-Regard

Participants’ sense of authenticity seemed rooted in an evolving self-knowledge and awareness. One participant (TO6), for example, notes, ‘*I knew in the back of my head that I was wrong and that I needed to acknowledge that I was wrong. I knew I was wrong and if I could not recognise that I would not have been my best-self’*. Their self-insight develops from discovering and learning from their inadequacies, to develop a sense of self-worth despite potential limitations as described by PO2, ‘*I first had to get to know myself as my personality, who I am, and then things fell into place*’ and affirmed in the words of TO3, ‘*Each person plays a part in the bigger picture. When I realised this, it became easier for me and I stopped competing with others. I concentrate on my own life and my own goals for myself and what I want to reach in my life’.* Similarly, TO1 emphasises her positive self-regard as an expression of feeling her best-self, ‘*You are your best-self when you are feeling good about yourself, that’s the honest truth. It can be that things are still negative, I still do not have the money that I needed. I still do not have that, but as long as from the inside I am clear and I feel strong and motivated and all that.’* A sense of best-self evolves in moving from self-insight to self-acceptance and ultimately to positive self-regard as evident in how PO2 values her strength, ‘*I believe that one will never know your own strength if you do not push yourself to find out who you really are.’* Ultimately, positive self-regard denotes valuing the self despite limitations and feeling positive and confident about the self, based on self-knowledge and insight. Self-regard forms the bedrock of a mature sense of self and enables women to emphatically understand others better as well, leading to a mindful relatedness.

#### Mindful Relatedness

In describing their sense of authenticity when dealing with conflictual interactions, best-self was expressed when participants felt they could handle conflict calmly and maturely because they could access a sense of compassion and understanding for the other. Cranton and Carusetta (2004) defined authenticity in how self-awareness is used to understand, care for and have better relationships with others. From a position of positive self-regard (noted above), TO2 continues to describe her capacity to understand and accommodate self-other differences, ‘*What I also realise is, you get different personalities … people with different backgrounds, different cultures, and those play a big role’.* PO1 and TO1 reflect similarly on being more tolerant and understanding of others, ‘*I just realised one day … she is also a human being, maybe she has her own issues that causes her to be the way she is’* (PO1), and TO1 states that ‘*Members of the public they tend to back up their [angry] drivers…not knowing you are there to protect them … Now members of the public are not aware of those things’.* Compassion is part of being authentic because it is an orientation that enables one to be true to the self, while having sympathy for all beings (McGrath, 2013). Compassion was further reflected in having sympathy with transgressors, without losing their sense of self, as was noted by PO5, ‘*I understand their [transgressors’] point also*’ and affirmed by PO4, ‘*We expect people to behave, but people are hungry, they want to have something as you are having something’.* Similarly, sympathy as a component of mindful relatedness is also reflected in PO6’s memory about how she calmed herself in a conflictual situation ‘*And then I hear, actually also with a sympathetic ear that, the person is actually not that informed’.* Ultimately mindful relatedness is founded on understanding and accommodating self-other differences through compassion, enabling participants to conduct themselves in an emotionally controlled manner when dealing with conflict in the workplace.

### Feeling Spiritually Congruent and Grounded

Participants narrate best-self experiences as rooted in feeling spiritually congruent and grounded. Their spiritual groundedness is therefore described in the experience of spiritual self-congruence and tapping into spiritual strength resources to help them cope when work circumstances are conflictual and challenging.

#### Spiritual Self-Congruence

Spiritual self-congruence reflects participants’ authenticity as rooted in a strong spiritual identity that is congruent to living according to and acting from spiritual values and norms. In reflecting on being her best-self, TO2 notes ‘*I will also link it [best-self] very strongly to my spiritual background, the fact that I am very spiritual and have the Lord in my life, because I have spiritual values’.* Their actions are driven from internalised spiritual values, such as serving others, ‘*Because what you are doing today to a person will be done to you one day’* (PO4). Their spiritual identity establishes a value-based benchmark reflecting how they ideally see themselves, and when they are not like that, they do not feel authentic. As noted by TO4, ‘*I talk to God…I say Lord…I am not like that. Take it away so we can go on’.* Similarly, TO1 reflects on her ideal self in a spiritual context, ‘*God talk to me, show me where I went wrong, show me where what I can do to better myself’.* Speaking about her best-self, according to TO6, God has helped her to know herself and change herself for the better, ‘*And I pray a lot for my temper … and dear Jesus must help me with that*. *I can see that I have changed a bit, not a bit, I have changed’.* It is in acting in congruence with their internalised values that participants experience a sense of authenticity. The internalised spiritual values of how one should be become part of the benchmark against which participants measure the self and especially the sense of being best-self, as denoted by TO1, *‘That is why as an individual you cannot speak out of anger… yes we have got contracts binding us, job descriptions binding us, but what does God say? What does the Bible say? What do the Ten Commandments say?’.* In experiencing authenticity as best-self, it appears that the mind is illuminated, and the self is elevated beyond self also by spiritual conceptions (Vaughan, 2002).

#### Tapping Into a Spiritual Source of Strength

Feeling spiritually grounded stands apart from spiritual self-congruence also strengthened by tapping into strength resources of a spiritual nature. Women expressed authenticity to the extent that they could trace their source of strength and energy to a higher power. They feel that they can only cope, especially given the nature of their work, by deriving strength from a higher power, as expressed by TO1, ‘*More especially if you are in this kind of work where you are on the line, … we need God’.* They lean on a spiritual source, especially in challenging situations over which they have no control. This is resolutely confirmed by PO2, ‘*I firmly believe that a person can do nothing on his own strength and that God brings things on your path and with it the necessary strength to get through it’.* Their experiences show how participants associate their best-self with a higher power helping them to deal with emotions of self and others. Being their best-self is not in and from themselves, but ‘*The Lord is the one who keeps a person going. One goes at night and pray and then tomorrow you feel alright again and then you go on again’* (PO5). Some participants expressed accessing or connecting with their higher power by attending church, praying and/or reading the Bible. Tapping into their higher power in this way is how they access the internal resources they need, such as strength and wisdom to handle problems in a way that leaves them with a sense of best-self. Hence, even when faced with pressures (Walsh and Vaughan, 1993), cognisance of the spiritual strengths at work within helps them remain aware of the positive aspects of their being (Zukav, 2001; Murray et al., 2004). Such aspects include how they feel when they do work that aligns with their passion and purpose (Waterman, 1993).

### Self-Actualisation in the Work Role

Participants’ experiences of best-self manifest in expressions of self-actualisation in the work role. Their self-actualisation is conceptualised here in expressing self-congruence with the work role, experiencing work as meaningful and in authentic self-expression.

#### Expressing Self-Congruence With the Work Role

Participants relate a sense of best-self to experiences reflecting a strong identification with their work role. Participants’ identification with their work role is evident in their describing a sense of congruence between self-values and their ability to express these at work. For TO5 this sense of being able to be herself in the work role, results in positive self-assessment, ‘*I was like myself there and I was very happy… I like to talk and I make people feel at ease’.* PO5 also relates to how in comparison to a previous work role, she now feels at home in her current role, making her happy to be a police woman, ‘*There I felt like a clerk … I did not feel like a police woman … it was so monotonous that I later developed half of a depression there … where I am now I’m very happy’.* In this sense, participants feel their best-self when they feel that the job affirms who they are and makes them feel good about themselves. Experiencing work as a place where they can live the person values that they hold in high regard leaves them with a sense of being their best-self, as noted by PO1, ‘*It [Police station] was really where I could express myself in my career by working with people, by building relationships, and my passion was working with people’.* Self-congruence in the work role is also reflected in descriptions of passion and enjoyment in relation to the work they do, as evident in the words of TO3, ‘*Somebody who is in a job and who does not love that job is actually more of a waste for the company and for himself’.* Similarly, TO2 notes, ‘*I fell in love with the occupation of a traffic officer and I enjoyed my work tremendously’.* In this way, several participants reflected on the positive emotions they experience because of a positive identification with their work role and the feeling that they can live the self-values they hold in high regard at work. This positive identification with the work role is further strengthened in that they find meaning through their work.

#### Experiencing Meaningful Work

Participants locate best-self experiences in feeling that through their work they are making a meaningful contribution to society. Some find meaning in serving others through their work, ‘*If I assist people and people are happy about what I’ve done for them then I feel I’ve done something; you know?’* (PO4). Similarly, PO6 describes how she found this occupation, meaningful and rewarding because it enabled her to help others, ‘*I could help people better…and the community in that instance’.* Making a meaningful contribution for participants resides in doing something of personal significance for which they have a passion. Participants note that ‘*When I am happy and I am at work, then I do my best, it’s as if you go that extra mile’*. (TO6). One participant, TO1, reflected on a sense of best-self by saying ‘*And you looked forward to going [to work], because when you do something for another person you feel good yourself’.* The meaning that participants derive from work stems from engaging with work activities resembling values that they consider to be important.

### Re-aligning to a Positive Way of Being

During the interviews, participants inadvertently narrated experiences of best-self in response to work-related situations of a challenging, frequently violent and conflictual nature. It seems that it is especially such emotionally challenging situations that elicit a need to return to a sense of best-self. In response to conflictual interactions, participants for example explain how they experience negative emotional charge which moves them away from feeling authentic. PO2 for example says, ‘*When you deal with difficult people who causes you to forget who you are, that brings out the ugly in you, is when I am no longer my best-self’*. The words of TO6 likewise reflect a sense of moving away from best-self when negative emotions are not regulated, ‘*When I am myself is when I am positive…because when you are not yourself you snap’.* TO1 also realises how negative emotional charge removes her from best-self, ‘*If I’m sad or there is something that is not making me happy, I’m not gonna perform my best-self’*. Reflecting on being emotionally charged in a work situation leads the women to recall consequent negative feelings and low self-affirmation. This helps them to recognise not having a sense of best-self and to activate thoughts and emotions to realign to a sense of best-self, as noted by TO6, ‘*Where not being my best-self almost got me into trouble, when my emotions controlled me a bit, I was a bit tough with a motorist … I am glad it happened to me so I can prevent it in the future, because it can cause big trouble if a person does things when your emotions overwhelm you’.*

Based on their knowledge of self (see theme 1, mature self), women seem able to identify and express their emotional reactions to daily work challenges appropriately, like stated by PO3: ‘*I became scared’;* TO6: ‘*Sometimes you can feel so down’*; PO5: ‘*I get so angry that I do not actually know what I must say, then I walk away’*; TO2: ‘*I was very nervous … you are exposed to the public, drunk people’;* and TO2*: ‘It is hurt … it is sadness, it’s a lot of things, which one must deal with’.* The ability to express thoughts, emotions, perceptions, stimuli and experiences as words is related to authenticity (Baer et al., 2004). When participants express their emotions, it enables them to activate their inner resources to deal with their negative emotions, and in a continued strive for positive affect, adjust to focus on positive and constructive responses. Women consequently engage coping thoughts to deal with the stressful situations (*cf.* Lazarus, 1993) and these thoughts are affirmative or positive (Kernis, 2003) realigning the women to their sense of who they are when they are their best-self. Through being more positive women feel more authentic, which is congruent to the emotive effect explained in the process of self-verification (Lehman et al., 2019). When narrating circumstances when they felt their best-self, they recall being optimistic, hopeful and confident, for example as stated by PO1, ‘*I always try to be positive … irrespective, because I always tell myself I am not here [at work] the entire day, let me do the best for the 12 h that I am here’.* Participants’ ability to recognise emotional demands and consequent negative emotional responses helps them to express their emotional responses and this is followed by active attempts to deal constructively with their emotions. This self-adjustment process is described here as realigning to a positive way of being, because at its essence it is the need for positive affect that is driving the adjustment process.

## Discussion

Women in law enforcement’s experiences of authenticity were explored from a best-self perspective, with the aim of constructing an understanding of when they experience a sense of best-self. The inquiry was approached from an existentialist, eudaimonic notion of authenticity, purporting the authenticity experience to denote a sense of best-self. Four themes were constructed from the data that constitute fundamental pillars in experiencing a sense of best-self. It is proposed that participating women in law enforcement, express a sense of best-self when they present with a mature sense of self, feel spiritually congruent and grounded, experience self-actualisation in the work role and realign to a positive way of being.

Best-self is experienced when participants express a mature sense of self. A mature sense of self is described as having a positive self-regard that is rooted in a consistent process of developing self-knowledge, accepting who you are and having a strong sense of self-worth and self-confidence. Self-worth or self-regard is essential for becoming a fully functioning person (Rogers, 1961) and is an essential component of a positive self-esteem (Cast and Burke, 2002). Yet, a mature self-esteem balances self-interest with responsibility to others (DuBrin, 2004). The mature sense of self thus extends positive self-regard to others by being mindful about and having compassionate tolerance for self-other differences. Embracing the self while also respecting others enables participants’ ability to negotiate and accommodate interpersonal differences, especially in a work context wherein they are consistently challenged with interactions of an aggressive and violent nature. Thus, a mature sense of self is based on a balanced and constructive self-other orientation that empowers the participants to cope with conflict and negative emotions in a constructive and self-empowered manner, leaving them feeling their best-self.

Secondly participants’ spiritual congruence and grounding seem fundamental to their sense of best-self. Exploring authentic being will unavoidably unearth facets of a spiritual nature (Hiles, 2000), especially when prompted by an approach to authenticity as being congruent to one’s best-self (Roberts et al., 2005). Women described being spiritually grounded as rooted in the experience of spiritual self-congruence, in deriving strength and coping from spiritual resources. They further describe being aware of the presence of a higher power in their lives and it is in living in congruence with this awareness, that they sense their best-self. Participants identified a Christian religion although religion was never part of a specific question in the interviews. None religion-specific aspects also influenced their spiritual grounding, such as living according to internalised values and beliefs. Authenticity from a best-self perspective highlights individuals’ extraordinary aspects (Roberts et al., 2005) that relate to their sacred internal being (Anderson and Braud, 2011) or higher power (Barton, 2009). From such being they form a spiritual identity (Poll and Smith, 2003) that grounds their behaviour (Suzuki, 2010), since tapping into this ‘ultimate being’ (Kilcup, 2016, p. 248) helps them to feel, think and behave in alignment with their higher power (Barton, 2009; Howard, 2009). Spirituality relates to belief systems involved in universal quests for meaning and purpose, regardless of religion or the absence of religion (Murray et al., 2004). When participants mirror their religious beliefs and values in their actions, they experience a sense of authenticity and in so doing they furthermore associate best-self with a strong spiritual identity. Thus, viewing authenticity from a spiritual congruence perspective enables participants to realign the self with the characteristics and values they hold in highest regard.

Authenticity as best-self, is also experienced when participants feel self-actualised in their work role. In the findings, self-actualisation in the work role firstly describes how participants identify with their work role and experience self-congruence as they are able to express and live the characteristics they value in themselves, in the work role. Self-actualisation relates to state authenticity in that it refers to being oneself or actualising the real self by developing the self to its highest potential (Cohen, 2008). Feeling affirmed because they can be themselves and live out who they are and the person characteristics they value in their law enforcement role, relates to what Glavas (2016) describes as doing work that is true to the self. Maslow’s conceptualisation of self-actualisation is based on the value ideals individuals integrate from societal norms of what is good and worthy (Cohen, 2008). Self-actualisation in this sense reflects an ideal self or in the context of this study, a notion of best-self. Self-actualisation was secondly related to the participants finding the work they do meaningful. Meaningful work reflects the individual’s subjective value ideal of balancing self-other interests and it is regarded in literature as the bedrock of self-actualisation (Bailey et al., 2019) and essential to being authentic (Martela and Pessi, 2018). Although meaning is subjectively constructed, the subjective evaluation of what is meaningful is inextricably linked to societal norms (Martela and Pessi, 2018). Ultimately work is regarded as meaningful, when it enables self-actualisation and brings the individual closer to the ideal self (Bailey et al., 2019).

Lastly, the participants describe their ability to realign to a positive way of being as characteristic of them being authentic from a best-self perspective. These include returning to ways of being that are optimistic and hopeful, while being associated not only with authenticity (Avolio and Gardner, 2005), but also with wellbeing and coping (Lazarus, 1993; Karademas, 2006). Feeling positive emotions is, however, not a given in the context where they work. Their related experiences are more probably based on an active process of constantly choosing to realign the self with thoughts, emotions and behaviour associated with best-self. Since possibilities of being authentic or inauthentic are both already within individuals, we are already what we want to become, so that actual *being* depends on which possibility we intentionally manifest (*cf.* Heidegger, 2010).

It seems that the conceptual array and disagreement around authenticity may, at its core, be related to the inability to define the internal referent, that is central to the verification process when trying to gain a sense of people’s authenticity experience. The idea of a singular true self or consistent personality does not hold in the context of social identity theory, which acknowledges a multifaceted self-concept that continues to develop as normally functioning people engage with and adapt to new and changing environments (Baumeister, 2019). It is utopic to operationalise the theoretical ideals of being completely authentic in any social context, wherein people change and are influenced externally (Ariza-Montes et al., 2017). A true self, real self, or a core self may be illusive to its own subject, whereas reflecting on one’s best-self may be more accessible and may offer insight into feeling authentic that is not always accounted for by only happiness and satisfaction (Smallenbroek et al., 2017). In response to the core interview question, participants inadvertently narrated experiences of self in response to work-related situations of a conflictual and challenging nature, which at first does not provide the in-the-moment feeling of happiness and satisfaction. It is in processing the event, evaluating and adjusting their actions and emotional responses that participating women find a state of being that they are satisfied and happy with, that is a sense of what they feel is their best-self. It is also in this process that the need to feel happy and content drives the strive to consistently work on the self, and in doing so it may sometimes, and even frequently, result in feeling happy. This experience as described in the findings reflect what the literature defines as state authenticity and aligns with eudaimonic identity theory, which posits that in constructing a sense of self, one is concerned with developing and demonstrating the best-self (Waterman et al., 2010). Being authentic is thus a consistent process of negotiating self-other expectations. This defines authenticity as a process akin to identity construction, but it does not exclude the ideal of finding happiness and satisfaction in work and in life. In fact, the hedonic notions of wellbeing seem intertwined in the broader context of eudaimonia as the need to realign to a positive way of being seems to be at the core of feeling good about the self in relation to others, how one lives according to your spiritual values and beliefs and how one finds congruence between the self and the work role. The findings provide a conceptual grounding for authenticity as a relational construct—the experience of self in relation to the self, to others, to a higher power and to work.

### Implications

This study emphasises the importance of establishing a sound conceptual orientation to the study of authenticity. As such, researchers should ground their conceptual understanding in a clear paradigmatic orientation, distinguishing either an existentialist or essentialist orientation, a hedonic and/or eudaimonic stance and favouring a state or trait perspective. More authenticity research is needed that takes an existentialist, eudaimonic orientation and regards authenticity as a consistent process of self-construction in view of having a best-self experience. Specifically for women, this may shed light on their identity work as a self-determined, self-constructed narrative, rather than an external ideal of consistently displaying feminine or masculine behaviours. Women should know that they can be authentic while growing their leadership style, while adjusting to work demands and role requirements in the workplace. They should know that they are the authors and judges of their authenticity experience. Pragmatically, to cope with work–life challenges and stressors and to enhance psychological wellbeing, support interventions should facilitate women’s authenticity by focussing on the development of a sense of best-self. Such a focus should entail working with authenticity as a relational phenomenon—the self is consistently constructed in relation to the self, to others, a higher power and to work. As such, working intra- and inter-personally to develop a mature sense of self (positive self-regard and mindful relatedness) is needed. Interventions should furthermore capitalise on developing spiritual resources and enabling women to express their spiritual values and ideals in how they relate in the work context. Lastly, developing women’s sense of self-actualisation in the workplace can be attained by developing a strong career identity and helping them establish meaning in their work endeavour.

## Conclusion

Developing authenticity is a relational identity construction process that helps women to cope, adjust and flourish in the workplace, in part because it is activated by challenges or stressors. It facilitates our concept of self, our interconnectedness with others and as a spiritual being and it enables self-actualisation in the work–role. Developing authenticity is a cyclical process of consistently returning to one’s sense of best-self and aids eudaimonic as well as hedonic wellbeing. The study contributes to our perceptions of authenticity and how authenticity is about becoming (or returning to) your best-self. It adds to the body of existing knowledge on authenticity by exploring women’s authenticity as a means of coping and flourishing in the law enforcement context.

## Data Availability Statement

The datasets presented in this article are not readily available because participants provided informed consent to use data for the purposes of this study. The data is not available for any other use. Requests to access the datasets should be directed to barnaha@unisa.ac.za.

## Ethics Statement

The study involving human participants were reviewed and approved by the relevant Research Ethics Review Committee in the College of Economic and Management Sciences of the University of South Africa (UNISA). The patients/participants provided their written informed consent to participate in this study.

## Author Contributions

RJ and AB contributed to the conception and design of the study and all sections in the manuscript and collaborated on the final findings in this manuscript. RJ did the fieldwork and a first level data analysis for her PhD in Psychology and contributed to revision and approval of submitted version. AB did a second level of analysis for this manuscript and final write up. All authors contributed to the article and approved the submitted version.

## Conflict of Interest

The authors declare that the research was conducted in the absence of any commercial or financial relationships that could be construed as a potential conflict of interest.

## Publisher’s Note

All claims expressed in this article are solely those of the authors and do not necessarily represent those of their affiliated organizations, or those of the publisher, the editors and the reviewers. Any product that may be evaluated in this article, or claim that may be made by its manufacturer, is not guaranteed or endorsed by the publisher.

## Footnotes

^1^We favour the spelling of best-self with a hyphen as presented by Roberts et al. (2005), as opposed to others who omit the hyphen such as Cable et al. (2013).
